# The Relation of Drinking Water to Health

**Published:** 1897-02

**Authors:** 


BUFFALO MEDICAL JOURNAL
A Monthly Review of Medicine and Surgery.

EDITORS:
THOMAS LOTHROP, M. D. - - WM. WARREN POTTER, M. D.

All communications, whether of a literary or business nature, should be addressed to the managing editor: 284 FRANKLIN STREET, BUFFALO, N. Y.

VOL. XXXVI. FEBRUARY, 1897. No. 7.

### THE RELATION OF DRINKING WATER TO HEALTH.

THE most important discussion to be brought before the Medical Society of the State of New York, January 26-28, 1897, is that on The relation of impure water to diseases and the cure and prevention of the latter. Important because it is timely—and should attract the attention of physicians as well as health authorities and sanitarians. The disease understood in this discussion is typhoid fever—perhaps as unnecessary a disease in highly civilised communities as any in our nosology. Typhoid fever is a disease due practically to carelessness, uncleanliness and the want of proper sanitary regulations. Its continued presence is as inexcusable in a municipality as vermin in a modern dwelling, and indicates a degree of shiftlessness and apathy not consistent with American views and energy.

Water-borne and water-bred, typhoid fever stands in close relation with the necessities of every-day life, and yet all our efforts, thus far, have been to cure the disease, instead of preventing its appearance. Many cities in which this disease is prevalent take their water-supply from navigable streams, at one time free from contamination, but becoming polluted by the refuse disposal of rapidly growing cities or villages situated farther toward the source of these same water-courses. Were the water properly treated, it would become as innocuous as if it were in its pristine purity and this is one of the lessons yet to be learned by many communities. In the rapid growth characteristic of many of our cities, the question of adequate water-supply became one of the great and pressing needs for early consideration, and natural sources were selected, more with the view of supplying *quantity* rather than

*quality* of water. Even if these sources had been of unquestioned purity, the improper disposal of the city's sewage, through the stupidity or carelessness of the proper officials, would have exposed the water to contamination and rendered it in time dangerous and polluted.

Besides the chemist and bacteriologist, the water department of every city, whose supply is liable to contamination, should have the services of an engineer, whose fame does not rest upon his theoretical knowledge and bureaucratic propensities, but upon his practical information of the laws of hydrology and hydrodynamics. Such an one, well versed in the hydrography of his locality, could almost prognosticate the condition of his water-supply and be an important aid to the city's health department. As such he would be able to render the same valuable services as does the local forecaster in meteorology, and give warning several days beforehand of marked changes in the quality of the water.

While the chemist and bacteriologist are only able to detect impurities after contamination, the engineer could foresee these changes and give sufficient warning, or attempt to overcome the impending contamination before the city mains and reservoirs are filled with the poisonous liquid.

Just what a pure supply means is hardly open to much debate and may be summed up in five propositions, as follows :

(1) That the water-supply of any city or village should not in any possible way be liable to pollution or contamination from the sewage of any other community.

(2) That the sewage of a city should not be emptied into any water-course not having a current of three to five miles per hour, and then the sewage entrance should be at a distance one mile or more from the intake.

(3) When the water-supply of any city or village is a navigable stream, the water should be sand-filtered before being pumped into the city reservoirs or water-mains.

(4) That for ordinary drinking purposes, the water should not be taken in its primitive or raw state, but be either filtered, boiled, or distilled and aerated.

(5) That not only chemical, but bacteriological, examinations of the water should be made, at least once weekly, to determine its character as a safe or dangerous water for domestic use, and where contamination is shown to exist, the services of an engineer be enlisted to detect, if possible, the cause and origin of such contamination.

We shall look forward to the publication of the discussion to be held at Albany with much interest, and with the expectation that it will lead to improvements on the lines indicated in these columns.

